# Long-term efficacy of imatinib mesylate in patients with advanced Tenosynovial Giant Cell Tumor

**DOI:** 10.1038/s41598-019-51211-y

**Published:** 2019-10-10

**Authors:** F. G. M. Verspoor, M. J. L. Mastboom, G. Hannink, R. G. Maki, A. Wagner, E. Bompas, J. Desai, A. Italiano, B. M. Seddon, W. T. A. van der Graaf, J.-Y. Blay, M. Brahmi, L. Eberst, S. Stacchiotti, O. Mir, M. A. J. van de Sande, H. Gelderblom, P. A. Cassier

**Affiliations:** 1 Radboud University Medical Center, Department of Orthopedics, Nijmegen, The Netherlands; 2 Leiden University Medical Center, Department of Orthopedics, Leiden, The Netherlands; 3 Radboud University Medical Center, Department of Operating Rooms, Nijmegen, The Netherlands; 4 Northwell Health/Monter Cancer Center and Cold Spring Harbor Laboratory, Department of Medical Oncology, Long Island, NY USA; 5 Dana Farber Cancer Institute, Department of Medical Oncology, Boston, USA; 6 Institut de Cancérologie de l′Ouest, Department of Medical Oncology, Nantes, France; 7 Peter Mac Callum Cancer Center, Department of Medical Oncology, Melbourne, Australia; 8 Institut Bergonié, Department of Medical Oncology, Bordeaux, France; 9 University College Hospital, Department of Medical Oncology, London, United Kingdom; 10 Radboud University Medical Center, Department of Medical Oncology, Nijmegen, The Netherlands; 11 Centre Léon Bérard, Department of Medical Oncology, Lyon, France; 12 Istituto Nazionale Tumori, Department of Medical Oncology, Milano, Italy; 13 Gustave Roussy Institute, Department of Medical Oncology, Villejuif, France; 14 Leiden University Medical Center, Department of Medical Oncology, Leiden, The Netherlands

**Keywords:** Outcomes research, Targeted therapies

## Abstract

Tenosynovial giant cell tumors (TGCT), are rare colony stimulating factor-1(CSF-1)-driven proliferative disorders affecting joints. Diffuse-type TGCT often causes significant morbidity due to local recurrences necessitating multiple surgeries. Imatinib mesylate (IM) blocks the CSF-1 receptor. This study investigated the long term effects of IM in TGCT. We conducted an international multi-institutional retrospective study to assess the activity of IM: data was collected anonymously from individual patients with locally advanced, recurrent or metastatic TGCT. Sixty-two patients from 12 institutions across Europe, Australia and the United States were identified. Four patients with metastatic TGCT progressed rapidly on IM and were excluded for further analyses. Seventeen of 58 evaluable patients achieved complete response (CR) or partial response (PR). One- and five-year progression-free survival rates were 71% and 48%, respectively. Thirty-eight (66%) patients discontinued IM after a median of 7 (range 1–80) months. Reported adverse events in 45 (78%) patients were among other edema (48%) and fatigue (50%), mostly grade 1–2 (89%). Five patients experienced grade 3–4 toxicities. This study confirms, with additional follow-up, the efficacy of IM in TGCT. In responding cases we confirmed prolonged IM activity on TGCT symptoms even after discontinuation, but with high rates of treatment interruption and additional treatments.

## Introduction

Tenosynovial giant-cell tumor (TGCT), historically known as pigmented villonodular synovitis (PVNS), is a rare, at times locally aggressive neoplasm affecting the joints or tendon sheaths in young adults. It is most common around large joints such as the knees, ankles and hips^1,2^. Known subtypes are localized and diffuse TGCT. The localized subtype comprises a single nodule and has a favorable course while the diffuse subtype involves the synovial lining as well as surrounding structures and is associated with a significant risk of recurrence (>50% depending on follow up times), despite being a benign neoplasm^2–4^. Metastatic forms have been described, but seem to occur very rarely^5,6^.

Surgical resection is the primary treatment for both subtypes. However, diffuse TGCT is difficult to remove completely and often requires a total synovectomy, or at times a joint replacement, or rarely even amputation^1,2,7^. In patients with extensive and/or recurrent TGCT, other available treatment modalities include radiation synovectomy^8^, external beam radiation therapy^9^, and cryosurgery^10^. Their therapeutic value has only been assessed in retrospective, in most cases single center series and their long term side effects and complications are poorly described^11^.

Recurrent TGCT is rarely lethal, but frequently becomes a debilitating chronic illness with substantial morbidity to the joints and quality of life impairment, caused by the disease itself and the multiple treatments^2,12^.

In TGCT, a neoplastic clone constitutes a subpopulation (2–16%)^13^ of cells that overexpress colony-stimulating factor-1 (CSF-1). A t(1;2) translocation that links the *CSF1* gene on chromosome 1p13 to the *COL6A3* gene on chromosome 2q35 has been described and is believed to be responsible for the overproduction of CSF1 by neoplastic cells^13,14^. Inhibition of CSF1/CSF-1 receptor (CSF-1R) signaling has shown efficacy in the treatment of locally advanced and recurrent diffuse TGCT^15–17^.

Imatinib mesylate (IM) inhibits the CSF-1R kinase among other kinases^17^. It has been shown that inhibition of CSF-1R by imatinib is competitive with ATP, with a Ki value of 120 nmol/L^18^. We have previously reported on the efficacy of IM in TGCT. In the present study we provide long term follow-up on these initial patients and data on 33 additional consecutive patients.

## Methods

This retrospective study was conducted at 12 referral centers across Europe (9 institutions), the United States of America (2 institutions), and Australia (1 institution). The file of all patients with locally advanced, recurrent or metastatic TGCT, treated with IM were reviewed. Patient information was extracted from individual patients’ files at each institution by the local investigators and was provided in an anonymous form for final analyses. Histopathologic examination was performed at center of origin by pathologists with extensive experience in mesenchymal tumors. Response was measured using version 1.0 of Response Evaluation Criteria in Solid Tumors (RECIST). Data were described using percentages for qualitative variables and medians with ranges for continuous variables.

Patients were not treated following a fixed regimen. The study protocol and retrospective analysis was approved by the Ethics Committee in Lyon (Committee for the Protection of Individuals, Sud-Est IV, Lyon, France – L10-153 dated 9 December 2010) and was carried out in accordance with the applicable rules concerning the review of research ethics committees. Patients provided written informed consent to treatment with ‘off-label’ medication, for research review and analysis of medical records. Treatment decision was left to the treating physician. The study was conducted in accordance with ethical requirements that differed per country. National investigators dealt with it according to standard practice. All 12 centers at which the study was carried out approved access to the data.

Survival was plotted using the Kaplan-Meier method. Progression-free survival (PFS) was calculated from the date IM was started to the date of disease progression or death. The time to treatment failure (TTF) was calculated from the date IM was started to the date it was stopped because of toxicity, disease progression, or death, whichever occurred first. For patients with a surgical resection or other additional therapy after treatment with IM, PFS and TTF were censored at the time of surgery. Disease specific survival was calculated from the date IM was started to the date of death due to TGCT. Symptomatic response was defined as improvement of pain and/or joint function in patients who had symptoms at baseline. All statistical analyses were performed using R version 3.4.0 (R Foundation, Vienna, Austria) with packages ‘ggplot2’, ‘rms’, and ‘survival’.

## Results

### Patients

A total of 62 patients with histopathologically proven TGCT treated with imatinib were identified, their main characteristics are described in Table 1. Briefly, median age at diagnosis was 39 (interquartile range (IQR) 31–53) years and 45 (IQR 36–56) years at start of treatment with IM, the majority of patients were female (N = 39, 63%), and the knee (N = 35, 56%) was the most commonly affected joint (Table 1). At start of IM treatment, three (5%) patients had biopsy proven metastatic disease, 15 (24%) locally advanced disease and 44 (71%) locally recurrent disease. Among patients with prior operations for TGCT (n = 47), the median number of prior operations was 2 (range 1–9), and the time since the last operation was 23 (range 1–192) months. Median follow up of all patients was 52 (IQR 18–83) months.

**Table 1:** Descriptive of diffuse-type TGCT patients receiving imatinib mesylate treatment.

	Patients N (%)
Total	62 (100)
Median age at diagnosis (IQR), yrs.	39 (31–53)
Median time from diagnosis to start IM (IQR), yrs.	3.5 (1–8)
**Sex**	
Male	23 (37)
Female	39 (63)
**Tumor location**	
Knee	35 (56)
Ankle	11 (18)
Hip	6 (10)
Foot	4 (6)
Shoulder	1 (2)
Elbow	1 (2)
Head and Neck	2 (3)
Wrist	2 (3)
**Surgery before start IM**	
None	15 (24)
1–2	24 (39)
3–4	13 (21)
>4	10 (16)
Median N of surgeries (range)	2 (1–9)
Median time since last surgery (range), mo.	23 (1–192)
**Disease status**	
Locally advanced	20 (32)
Recurrence after surgery	39 (63)*
Metastatic disease	3 (5)

Abbreviations: TGCT = Tenosynovial Giant Cell tumor, IM = imatinib mesylate, N = Number of patients, mo = months, yrs = years. *One of the locally recurrent patients progressed to metastatic disease.

### Treatment efficacy

Sixty-one patients received 400 mg and one patient received 600 mg IM daily, all as a single dose/day. The 3 patients with metastatic disease at treatment start progressed rapidly on IM and were excluded from further analysis. One other patient with metastatic disease after multiple surgical treatments and IM, was excluded for further analyses too, leaving 58 patients for the rest of the analysis.

**Figure 1:**
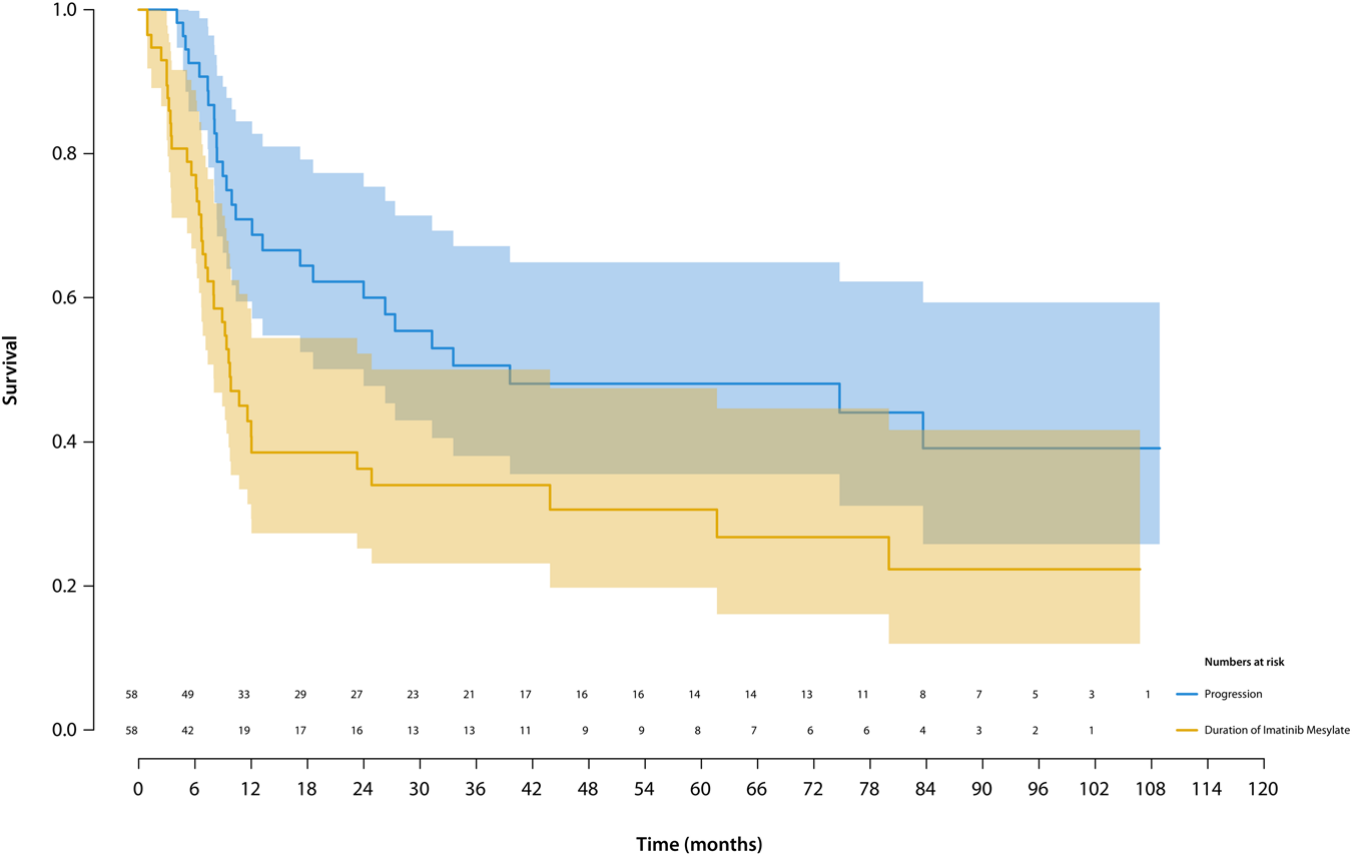
Kaplan–Meier survival curves showing the duration of imatinib mesylate treatment (yellow line) and progression free survival (PFS; blue line) in patients with locally advanced or recurrent diffuse-type TGCT. PFS was calculated from the date imatinib mesylate was started to the date of disease progression or death. The shaded areas are 95% confidence intervals (CI). Over half of the patients discontinued IM within a year. The overall PFS after 5 years was ~50%.

Median duration of IM treatment was 9 (IQR 5–27) months. At last follow-up, the majority of patients (n = 38; 66%) had discontinued treatment. Seventy-seven percent (95% CI 67–89), 41% (95% CI 29–57) and 36% (95% CI 25–52) of patients were still on IM after 6-, 12- and 24-months, respectively (Fig. 1). The treatment failure-rate was 18% (95% CI 6–29) after 12 months.

Response could not be assessed in 3 patients, two of which were lost to follow-up and one who discontinued early due to febrile neutropenia, leaving 55 patients with locally advanced or locally recurrent TGCT assessable for response. Seventeen patients (31%; 95% CI 19–43) had a RECIST-defined response, including 2 (3%) patients with a complete response. The median time to best response was 6 (range 1–23) months.

Forty of 51 patients (78%) reported symptom improvement (Table 2), including 14 of 15 patients with radiological response (CR or PR). Among patients with radiological stable disease (SD), 22 of 30 patients (73%), for whom data was available, had symptom improvement.

**Table 2:** Summary of imatinib mesylate efficacy in patients with locally advanced or recurrent diffuse-type TGCT.

Parameter	Patients N (%)
RECIST best response*	
Complete remission	2 (4)
Partial response	15 (27)
Stable disease	36 (65)
Progressive disease	2 (4)
Overall response rate	17 (31)
Rate of disease control	53 (96)
Symptomatic response	40 (78)**
Median IM treatment duration (IQR), mo.	9.3 (5–26)
Median PFS (IQR), mo.	18 (8–55)

Abbreviations: TGCT = Tenosynovial Giant Cell tumour, IM = imatinib mesylate, N = Number of patients, mo = months, yrs = years, IQR = inter quartile range.

Overall response rate includes complete remission and partial response; Rate of disease control includes complete remission, partial response and stable disease; Symptomatic response was indicated as present or not (40/51 = 78%). Metastatic patients (n = 4) were excluded.

*N = 3 RECIST best response not available; **N = 9 symptomatic response not available.

The 1-, 2- and 5-years overall PFS, metastatic patients (N = 4) excluded, was 71% (95% CI 60–85), 60% (95% CI 48–75) and 48% (95% CI 36–65) respectively(Fig. 1).

### Follow-up

**Figure 2:**
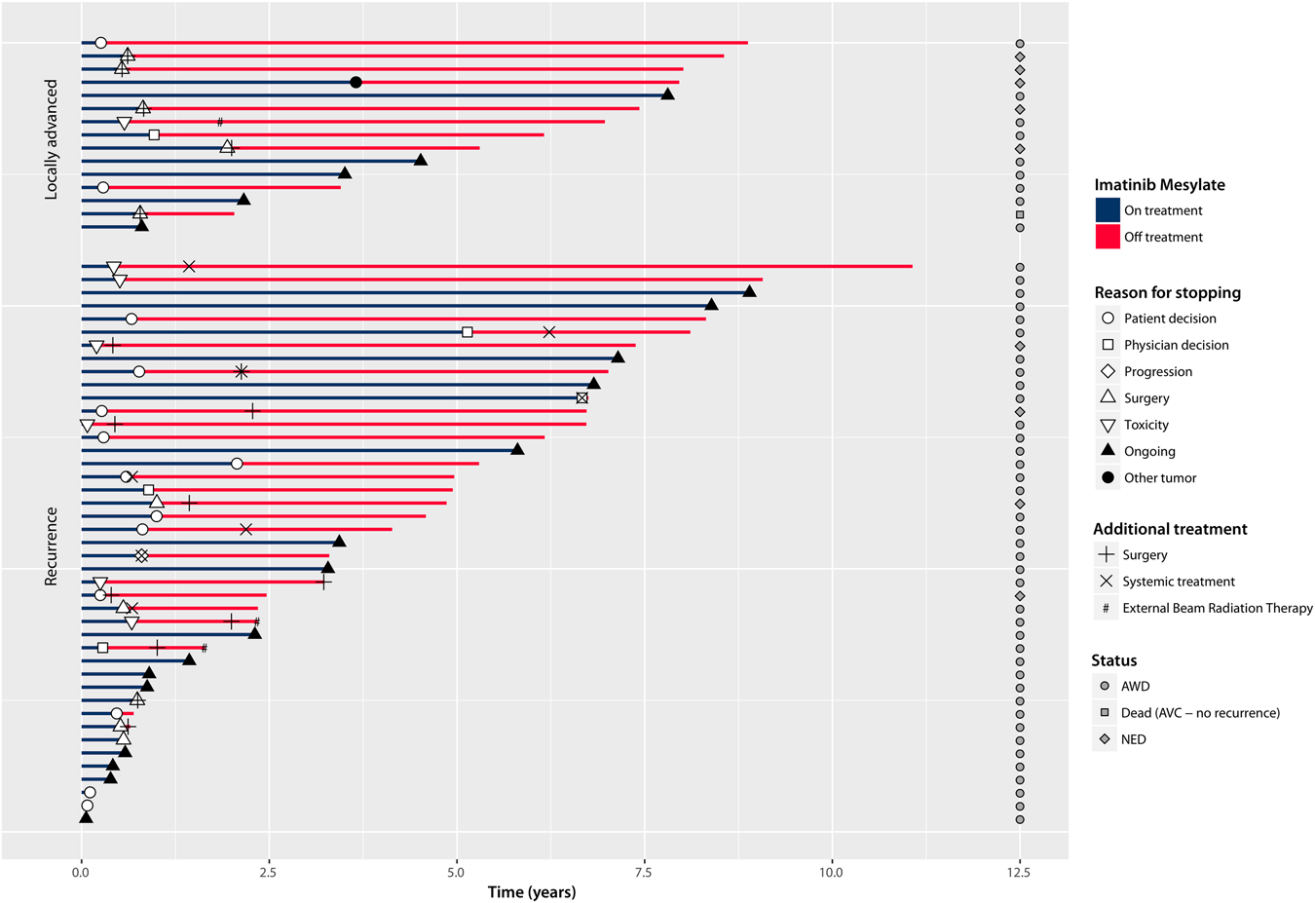
Response and follow up of imatinib mesylate in patients with locally advanced or recurrent diffuse-type TGCT. NED = No evidence of disease, AWD = Alive with disease.

Overall 38/58 patients (66%), metastatic patients (N = 4) excluded, eventually discontinued IM after a median of 7.0 (range 1–80 months). The most common reason for treatment discontinuation was patient decision to stop (n = 14, which possibly reflect low grade chronic toxicity), followed by planned surgery (n = 10), toxicity (n = 7), physician’s decision (n = 5) and progression (n = 1). One patient discontinued IM because of the diagnosis of another tumor requiring therapy. Among the 27 patients who discontinued treatment for reasons other than surgery or progression, progression (either radiological progression or requirement for another line of therapy – i.e. surgery, other medical therapy or radiotherapy) eventually occurred 17 patients after a median of 12 (range 4–84) months, while 10 patients never progressed (nor required additional therapy) after a median follow-up to 78 (range 1–109) months, suggesting that IM was able to provide prolonged symptomatic relief at least in a proportion of patients. Detailed information on each patient is presented in Fig. 2.

### Safety

Forty-five of 58 patients (78%), metastatic patients (N = 4) excluded, reported at least one adverse event with IM. The most common adverse events were edema (N = 28, 48%), fatigue (N = 29, 50%), nausea (N = 21, 34%) and skin rash/dermatitis (N = 7, 12%), mostly grade 1–2 (89%). Additional grade 1–2 complaints were diarrhea, reflux, auditory hallucinations, conjunctivitis, sexual impairment, asthenia, alopecia, cramps and dyspnea. Five (11%) patients had grade 3–4 toxicities, including neutropenia, acute hepatitis, facial edema, skin toxicity and fatigue (Table 3).

**Table 3:** Main toxicities associated with imatinib mesylate and reasons for discontinuation, metastatic patients excluded.

	Patients N (%)	
**Variable**	**All grades**	**Grade 3–4**
Edema/fluid retention	28 (48)	1 (2)
Fatigue	29 (50)	1 (2)
Nausea	20 (34)	
Skin rash/dermatitis	7 (12)	2 (3)
Other*	15 (26)	3 (5)
**Treatment status**		
Continued on IM	20 (34)	
Stopped IM	38 (66)	
**Reason for stopping**		
Progression	1 (2)	
Toxicity	7 (12)	
Surgery	10 (17)	
Patient choice	14 (24)	
Physician decision	5 (9)	
Other tumor	1 (2)	

IM = imatinib mesylate, N = Number of patients. Forty-five (78%) patients reported at least one adverse event with IM. *Other grade 1–2 complaints were diarrhea, reflux, auditory hallucinations, conjunctivitis, sexual impairment, asthenia, alopecia, cramps and dyspnea. Five (11%) patients had grade 3–4 toxicities, including neutropenia, acute hepatitis, auditory hallucinations.

## Discussion

To our knowledge, this retrospective study provides the largest case series, with long follow-up, of patients with locally advanced, recurrent or metastatic diffuse-type TGCT treated with IM. We confirmed that IM has activity in TGCT with an overall response rate of 31% in patients with locally advanced/recurrent TGCT. Interestingly all patients with metastatic TGCT progressed on IM, suggesting that metastatic TGCT is either a different disease or loses its dependency on the CSF1/CSF1R axis during malignant transformation. The main issue, is the drop-off rate, with more than half of the patients discontinuing therapy within a year of therapy (59%; 95% CI 29–57), in most cases for unclear reasons (patients decision, physician’s decision) suggesting an unfavorable efficacy/toxicity balance. Eleven percent of patients reported grade 3–4 toxicities, which is consistent with the rates reported with IM for adjuvant gastrointestinal stromal tumors (GIST) or chronic myeloid leukemia (CML)^19–22^.

To date, surgical resection remains the treatment of choice for diffuse-type TGCT, but is associated with high recurrence rates and multiple additional surgeries^11^. It is challenging to balance between increased morbidity of multiples or invasive surgeries^12,23^, alternative therapeutic options, and daily symptoms of the tumor. A more aggressive resection or other multimodality treatments, such as external beam radiation therapy, radiosynovectomy and cryosurgery, may adversely affect joint function, quality of life and development of osteoarthrosis, which, given the young age group, are relevant factors^2,24^. This would justify a less invasive approach, using systemic therapy, provided those are associated with tumor shrinkage and, most importantly, symptomatic improvements^25^.

In the present study, age, localization and gender distribution were consistent with the literature^10,24,26^. The extent of disease in our patient group is emphasized by a disease specific survival of 90% including four metastatic patients and 49% of patients had three or more surgeries before start IM. Similar to previous case-series, we calculated a 1- and 5-years PFS of 71% and 48%, metastatic patients excluded, respectively^10,24,26^. Because of heterogeneity of patients and a variety of treatments, it is debatable to compare these numbers.

The overall response rate appears higher compared to nilotinib 6% (95% CI unknown), a different tyrosine kinase inhibitor, with similar potency against CSF1R^27^. Our overall response rate 31% (95% CI 19–43, metastatic patients (N = 4) excluded) was consistent with our previous report on the short term results of IM 19% (95% CI 4–34) with similar disease control rate (96% versus 93%)^17^. In the present study, 38 (66%) patients discontinued IM; 14 (37%) without subsequent treatment, of which ten patients had stable disease at final follow up. Of the 38 patients who discontinued IM, 21 patients (55%) discontinued IM for toxicity or non-specific medical reasons. 13 (62%) out of these 21 patients eventually progressed. Both stable and progressive patients can be a result of discontinuing IM treatment or the natural course of disease.

Newer, more specific inhibitors of CSF1R, currently only available in trial-setting such as emactuzumab (RG7155)^28^, pexidartinib (PLX3397)^15,29,30^, and cabiralizumab^31^ (FPA008, Five-Prime), have shown promising clinical activity on similar groups of diffuse TGCT patients in prospective clinical studies with more formal criteria and timelines for response assessment than this retrospective series. Emactuzumab (N = 29)^16^ had an overall response rate of 86% (two patients with a complete response) and a rate of disease control of 96%, including a significant functional and symptomatic improvement (median follow up 12 months). Pexidartinib showed (N = 23)^15^ an overall response rate of 52% (all patients had a partial response) and a rate of disease control of 83%. At ASCO 2018 results of a pexidartinib placebo controlled, phase 3 study showed a significant improved overall response rate (39.3% vs 0%) and PROMIS physical function (4.06 vs 0.89), after a median 6 months follow up^29^. The preliminary results with cabiralizumab (N = 22) are consistent, with radiographic response and improvement in pain and function in five out of 11 patients^31^. However, long term efficacy data have not yet been reported with these newer agents.

Virtually all patients treated with IM for either CML or GISTs, experience^32^ at least one mild or moderate adverse effect (grade 1–2). Toxicities of IM are determined by the disease stage and the doses used, advanced disease and higher doses result in more frequent and severe toxicities. Most side effects occur early in the course of treatment and tend to decrease in frequency and intensity in time^32^. We consider a 10–15% rate of grade 3–4 toxicities in a generally benign but locally aggressive disease, such as diffuse TGCT, too high. Only 22% of patients did not experience any side effects.

Although target anti-cancer therapies are described as ‘well tolerated’, the perception of tolerability may vary in the context of a, most often, benign condition. Understanding, monitoring and managing the side effects will be important to optimize systemic therapy for patients with TGCT.

Discontinuation of treatment due to toxicities was seen for IM (this series), emactuzumab^15^ and pexidartinib^16^ in 12%, 20% and 9% patients, respectively. TGCT patients might be less willing to cope with adverse event-related and study-related procedures. Here, we report prolonged clinical benefit and symptomatic relief, even after discontinuation of treatment. A similarly persistent effect was observed with monoclonal antibodies and more specific CSF1R tyrosine kinase inhibitors^25^. This suggest that intermittent treatment administration may be an option to improve long term tolerability.

The place of systemic treatment in a benign, locally aggressive disease, such as TGCT, and how to optimally deliver this treatment, remains unclear. More specifically, the role of CSF1R inhibitors in the peri-operative setting still needs to be explored: the number of patients who underwent operation after IM in our series is too low to draw any conclusions. Despite limitations related to its retrospective nature, this study adds to the knowledge of targeting the CSF-1/CSF-1R pathway in patients with TGCT. An optimal treatment strategy should be developed for the patient group that benefits most from systemic therapy. The combination of a short period of treatment and the durable effect after discontinuation, should be pursued. It is challenging to maintain compliance for years, especially with, even “minor”, toxicities, in the context of a non-life-threatening disease.

So far, less or more specific tyrosine kinase inhibitors have been tested in selected patients who had inoperable, progressive, or recurrent disease^15–17,30,31,33^. These selected individuals represent a small group of severely affected patients that are a part of a much larger, often less complicated group of patients. The patients who would benefit most from systemic therapy should be identified on the basis of molecular tumour features sensitive to that specific treatment^34^. In addition, recently an objective MR imaging-based TGCT severity classification has been developed^35^. The proposed severity classification may be helpful to identify the more aggressive TGCT subtypes eligible for systemic therapy or trials for novel agents.

A limitation of all, including this, clinical TGCT studies is the lack of a control group and the absence of specific and validated patient-reported outcome measures to document treatment-induced symptomatic, functional and economic (back to work) improvement^16^. Quality of life and functional forms should be implemented. These measures are critical endpoints in demonstrating clinical relevance and impact of treatments for benign diseases in which death is not a relevant outcome variable^36^. Clinical benefit necessitates objective measures to correlate with tumor reduction.

## Conclusion

Identification of a biologic aggressive subgroup of diffuse TGCT, at risk of increased surgical morbidity or recurrent disease, should aid to decide which patients benefit most of systemic treatments. With the advent of more potent CSF-1R inhibitors, such as emactuzumab, pexidartinib and cabiralizumab, the role of IM in extensive TGCT might weaken, but may be balanced by the favorable safety profile of IM. Availability of these new compounds, both in terms of registration and reimbursement, will ultimately define the prescribed drug in daily practice^37^.
